# Elevated Stratifin promotes cisplatin-based chemotherapy failure and poor prognosis in non-small cell lung cancer

**DOI:** 10.1016/j.omto.2021.07.005

**Published:** 2021-07-21

**Authors:** Yu-Shui Ma, Li-Kun Hou, Shi-Hua Yao, Ji-Bin Liu, Xue-Chen Yu, Yi Shi, Xiao-Li Yang, Wei Wu, Chun-Yan Wu, Geng-Xi Jiang, Da Fu

**Affiliations:** 1 Department of Nuclear Medicine, Shanghai Tenth People’s Hospital, Tongji University School of Medicine, Shanghai 200072, China; 2 Cancer Institute, Affiliated Tumor Hospital of Nantong University, Nantong 226631, China; 3 Department of Pathology, Shanghai Pulmonary Hospital, Tongji University School of Medicine, Shanghai 200433, China; 4 Department of Thoracic Surgery, Navy Military Medical University Affiliated Changhai Hospital, Shanghai 200433, China; 5 Department of Mathematics, Statistics, and Computer Science, Macalester College, Saint Paul, MN 55105, USA

**Keywords:** SFN, NSCLC, GEO database, ACT, prognostic biomarker

## Abstract

Drug resistance is a key factor in the treatment failure of clinical non-small cell lung cancer (NSCLC) patients after adjuvant chemotherapy. Here, our results provide the first evidence that eukaryotic translation initiation factor 2b subunit delta (EIF2B4)-Stratifin (SFN) fusion and increased SFN expression are associated with chemotherapy tolerance and activation of the phosphatidylinositol 3 kinase/v-akt murine thymoma viral oncogene (PI3K/Akt) signaling pathway in NSCLC patients, suggesting that SFN might have potential prognostic value as a tumor biomarker for the prognosis of patients with NSCLC.

## Graphical abstract

**Figure:**
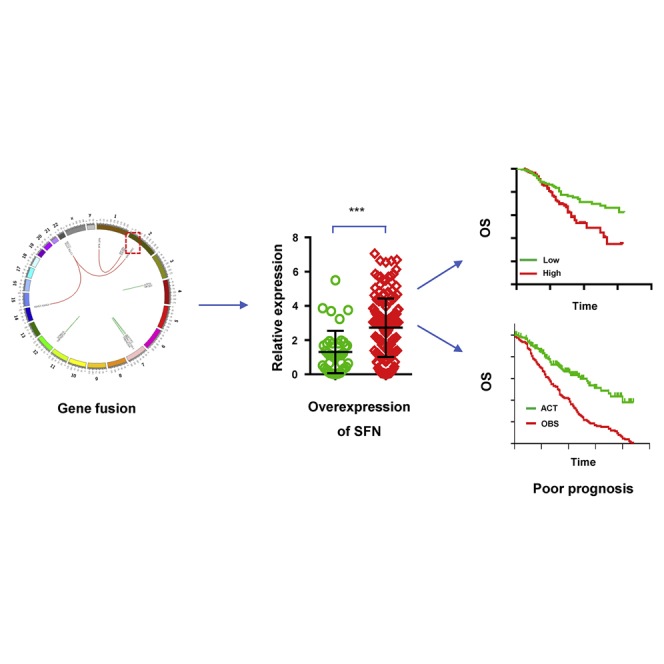

## Introduction

Lung cancer is characterized by high morbidity and mortality and is considered to be one of the most life-threatening cancers in the world.^1^ Despite advances in surgical techniques, molecular targeted therapies, immunotherapies, and more prevalent screening to diagnosis tumors earlier, the recurrence rate of non-small cell lung cancer (NSCLC) is reported to be as high as 30%–70%.^2^

In order to reduce the recurrence risk of NSCLC, adjuvant chemotherapy (ACT) is usually performed after surgery.^3^ Cisplatin-based ACT is a first-line, DNA damage-inducing, chemotherapeutic regimen acceptable for patients with pathological stage II and stage IIIA NSCLC that has been completely resected.^4^ However, drug resistance that occurs after ACT reduces its efficacy and is a key factor in the treatment failure of clinical NSCLC patients.

In this study, we explored Stratifin (SFN) expression and gene fusion in NSCLC patients after chemotherapy.

## Results

**Figure 1:**
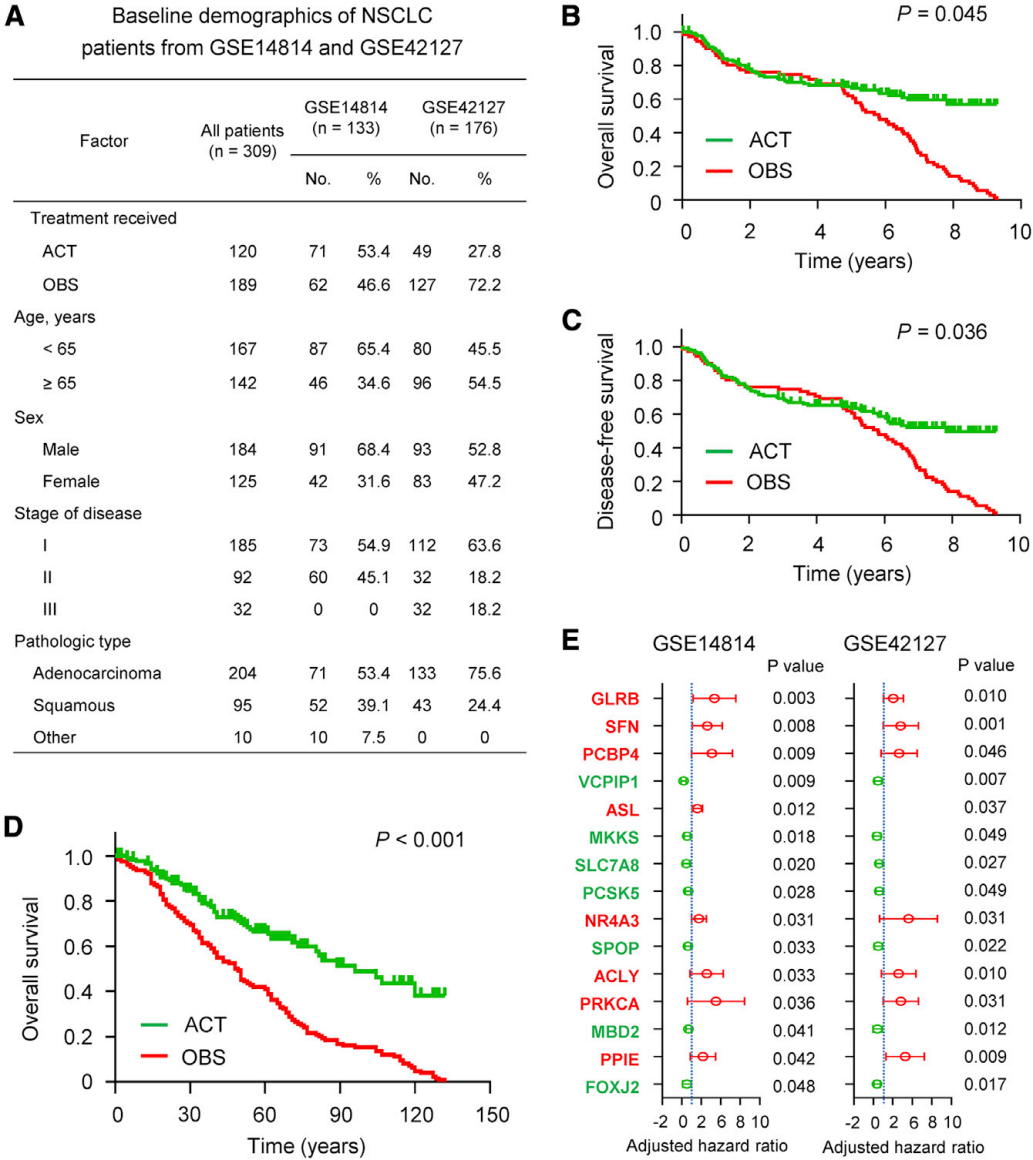
Analysis of the clinical significance of ACT and OBS in NSCLC using two GEO databases (A) Demographic information for NSCLC patients from the GSE14814 and GSE42127 datasets. (B and C) Kaplan-Meier survival analysis was used to evaluate the prognostic value of ACT in NSCLC from the GSE14814 dataset for overall survival (OS, B) and disease-free survival (DFS, C). (D) Kaplan-Meier survival analysis was used to evaluate the prognostic value of ACT in NSCLC from the GSE42127 dataset for OS. (E) Cox regression model analysis for prognosis based on various gene expression in NSCLC patients after ACT from the GSE14814 and GSE42127 datasets.

To investigate potential gene expression that acts as prognostic biomarkers for NSCLC patients under adjuvant chemotherapy, we evaluated the gene expression profiles of patients that received cisplatin-based ACT and controls using the Gene Expression Omnibus (GEO) database *in silico*. Figure 1A includes the basic information of 309 NSCLC patients from GSE14814 and GSE42127 datasets. As expected and consistent with previous studies,^4^ NSCLC patients with ATC from the GSE14814 dataset had significantly increased overall survival (OS; hazard ratio [HR] = 0.51 [0.48, 0.67], p = 0.045; Figure 1B) and disease-free survival (DFS; HR = 0.63 [0.58, 0.72], p = 0.036; Figure 1C). Furthermore, NSCLC patients from the GSE42127 dataset treated with ACT also displayed a significantly increased OS (HR = 0.44 [0.41, 0.52], p < 0.001; Figure 1D), which suggested that NSCLC patients benefit from ACT.

Next, we investigated the gene expression profile in patients receiving ACT treatments from both the GSE14814 and GSE42127 datasets. Gene expression profiling was conducted on mRNA from 133 frozen JBR.10 tumor samples^4^ and 176 frozen tumor tissues from the GSE14814 dataset. We found an expression signature from 15 genes that separated ATC patients into high-risk and low-risk subgroups with significantly different survival rates (Figure 1E). As shown in Figure 1E, the genes in red were upregulated and exhibited a negative relationship with patient prognosis, with higher expression resulting in a worse prognosis. The genes shown in green were downregulated and had a positive relationship with patient prognosis, with higher expression resulting in a better prognosis (Figure 1E).

**Figure 2:**
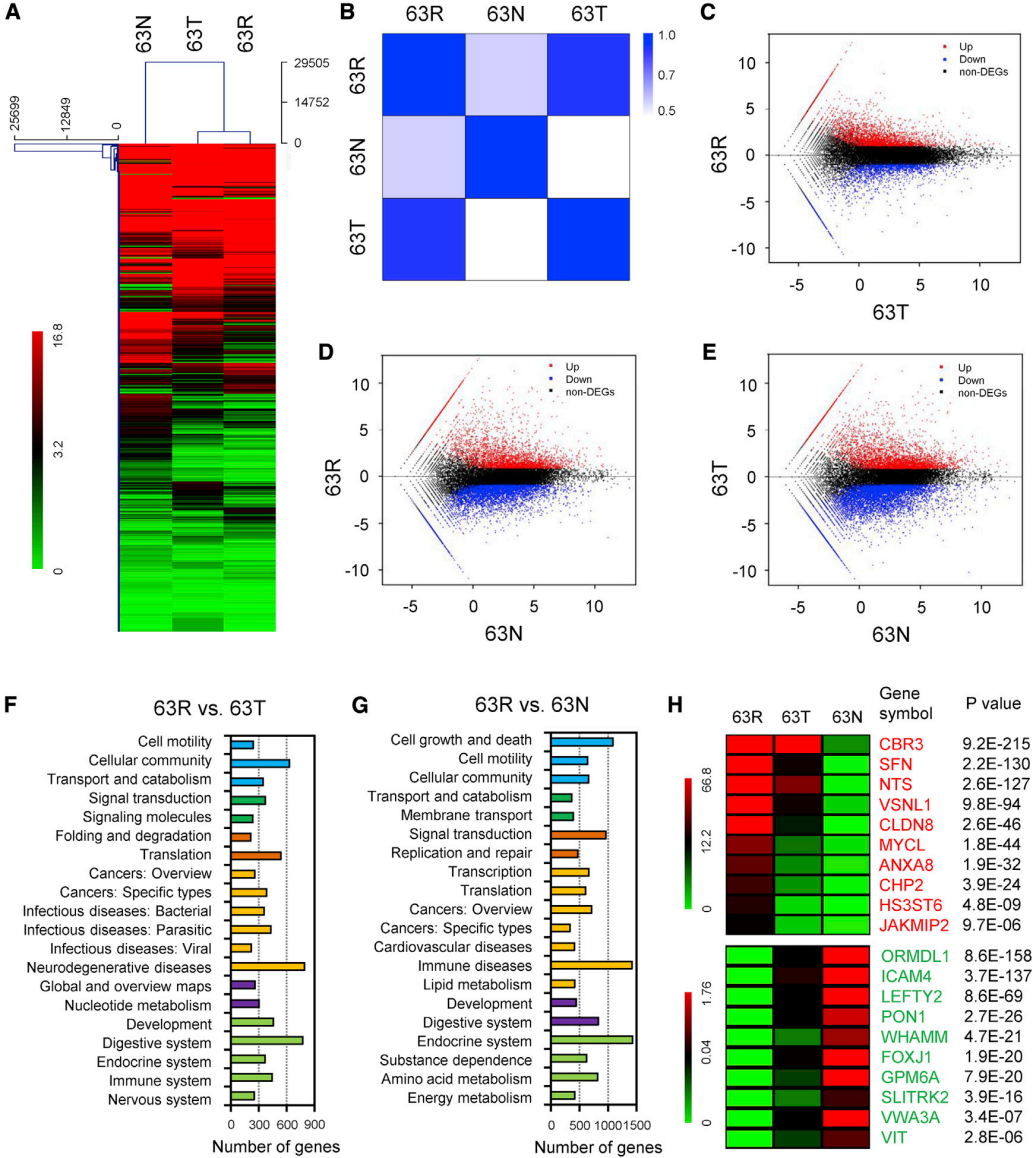
WTS analysis of a NSCLC triplet set (A) Clustered analysis of dysregulated genes (fold change [FC] ≥ 2 or ≤ 0.5, p < 0.01) from a NSCLC triplet dataset including adjacent non-cancerous lung tissue (63N), primary NSCLC tissue (63T), and recurrent lung cancer tissue after ACT (63R). (B) Correlation analysis of dysregulated gene expression among 63N, 63T, and 63R. (C–E) MA Plot for the gene expression microarrays of 63R versus 63T (C), 63R versus 63N (D), and 63T versus 63N (E). (F and G) KEGG analysis of dysregulated genes (FC ≥ 2 or ≤ 0.5, p < 0.01) in 63R versus 63T (F) and 63R versus 63N (G). (H) Heatmap plot of the 10 most upregulated and downregulated mRNAs with the greatest FC from the microarray data of 63N, 63T, and 63R. Red, upregulation; green, downregulation.

To investigate the genes that correlated with a worse NSCLC prognosis after cisplatin-based ACT, we obtained a triplet set from a NSCLC patient with recurrence (normal lung tissue [63N], primary tumor samples without treatment [63T], and recurrence focus tissues after ACT [63R]) for whole-transcriptome sequencing (WTS) analysis. We conducted hierarchical clustering analysis of RNA expression in a triplet set of a NSCLC patient using MEV4.7.1 software (Figure 2A).

Unsupervised hierarchical clustering and correlation analysis of the expression data showed that the primary cancer tissue and recurrent tissue had closely related expression profiles when compared to para-tumor normal tissue, suggesting clonal and genetic similarity for the pairs (Figures 2B–2E). We identified a total of 1,289 genes that were significantly changed in 63R as compared to the 63T or 63N groups. Kyoto Encyclopedia of Genes and Genomes (KEGG) pathway analysis of these differentially expressed gene patterns revealed several enrichment-related pathways, including cell growth and death, cell motility, cellular community, transport and catabolism, and signal transduction (Figures 2F and 2G). Figure 2H shows a heatmap plot of the top 10 upregulated and downregulated mRNAs with the greatest fold change from the microarray data of 63N, 63T, and 63R. Among them, SFN was found to be upregulated in both GSE14814 and GSE42127 datasets and was also found to significantly upregulate in recurrent NSCLC patient tissue when compared in para-tumor normal tissue and primary cancer tissue.

**Figure 3:**
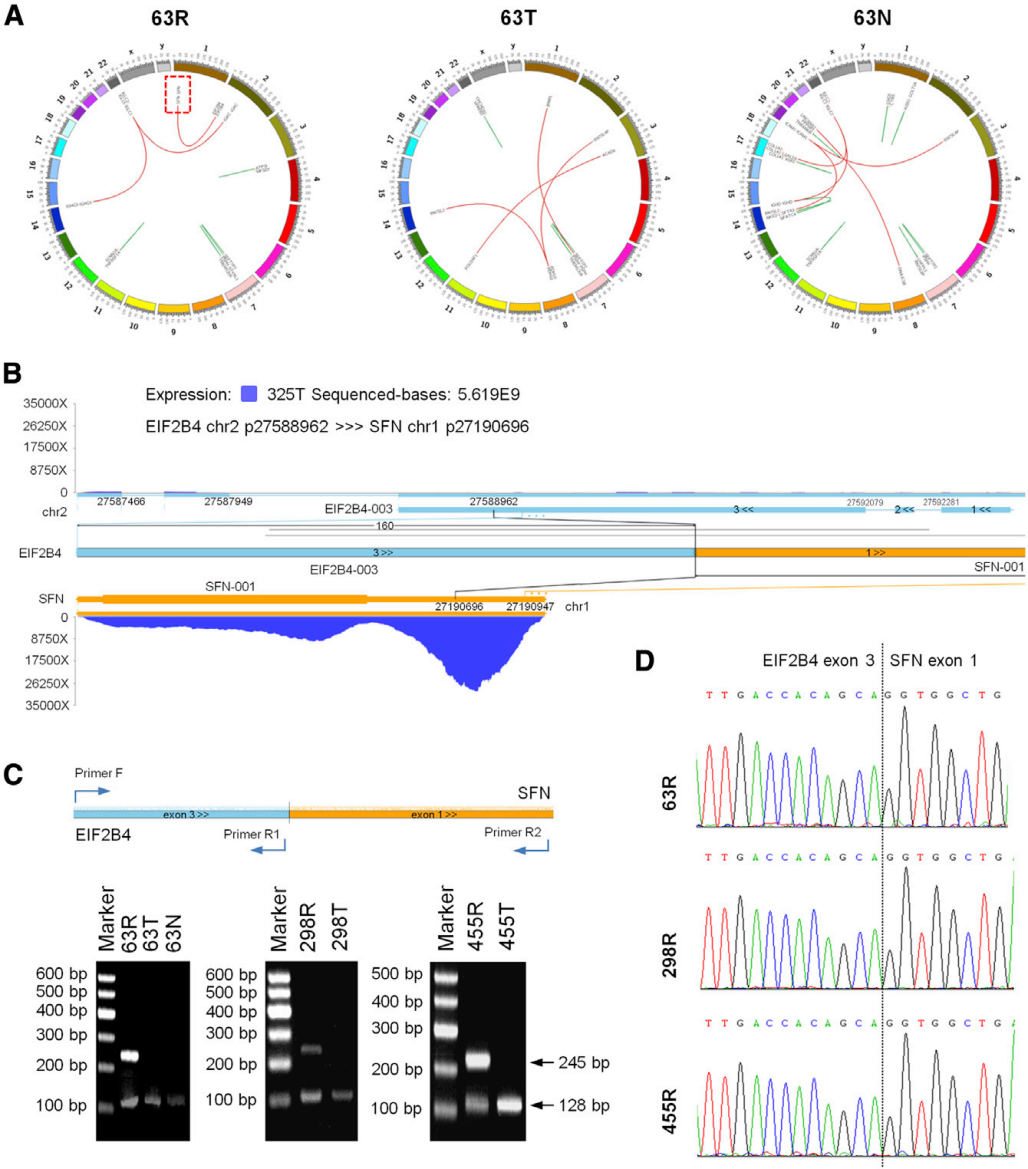
Gene fusion analysis in NSCLC patients after ACT (A) Circos plots of 63N, 63T, and 63R. Intrachromosomal rearrangements (inner circle green) and interchromosomal rearrangements (inner circle red). (B) Rearrangement of chromosome 2 in 63R produces the EIF2B4-SFN fusion. (C and D) RT-PCR amplification (C) and Sanger sequencing validation (D) of the EIF2B4-SFN fusion in 3 paired NSCLC patient samples after ACT and primary NSCLC samples.

Furthermore, we found that the N terminus of eukaryotic translation initiation factor 2b subunit delta (EIF2B4) was fused to the C terminus of SFN (Figures 3A and 3B). To verify the fusion discovered in WTS, we then performed a RT-PCR reaction (Figure 3C) and Sanger sequencing (Figure 3D) and the results of gene fusion were further confirmed. We then identified two recurrent cases, 298R and 455R, in 62 recurrent NSCLC samples after ATC that harbored an EIF2B4-SFN fusion, which resulted from an intra-chromosomal rearrangement that has never been described before (Figures 3C and 3D).

**Figure 4:**
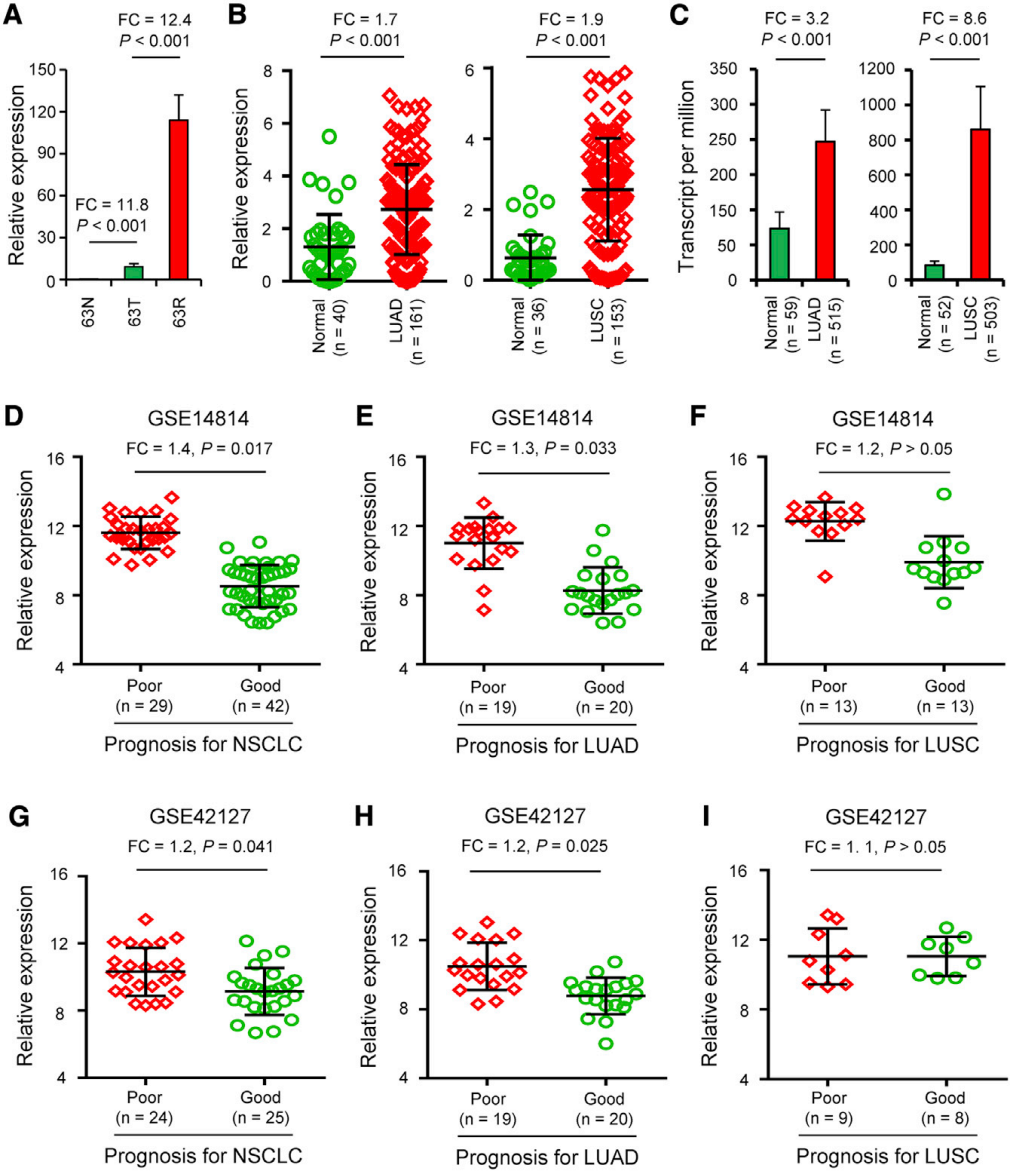
Expression and clinical significance of SFN in normal lung and NSCLC tissues (A) The expression levels (log2 median-centered ratio) of SFN in 63N, 63T, and 63R were analyzed. (B) The SFN expression in NSCLC samples (LUAD, n = 161; LUSC, n = 153) and adjacent non-tumor tissues (LUAD, n = 40; LUSC, n = 36) was analyzed using qPCR analysis. (C) The SFN expression in NSCLC samples (LUAD, n = 515; LUSC, n = 503) and adjacent non-tumor tissues (LUAD, n = 59; LUSC, n = 52) from TCGA dataset was analyzed.The expression level of SFN in 29 NSCLC, 19 LUAD, or 13 LUSC patients with poor prognosis (OS status: dead) and 42 NSCLC (D), 20 LUAD (E) or 13 LUSC (F) patients with good prognosis (OS status: alive) from the GSE14814 dataset. (G-I) Expression level of SFN in 24 NSCLC, 19 LUAD, or 9 LUSC patients with poor prognosis (OS status: dead) and 25 NSCLC (G), 20 LUAD (H), or 8 LUSC (I) patients with good prognosis (OS status: alive) from the GSE42127 dataset.

To further confirm the results of the WTS and *in silico* gene expression study, we analyzed and validated the expression of SFN in the triplet set of the NSCLC patient (Figure 4A) and large clinical samples using quantitative PCR (qPCR) analysis (Figure 4B). A total of 314 NSCLC tumor specimens (including 161 lung adenocarcinomas [LUADs] and 153 lung squamous cell carcinomas [LUSCs]) and 76 normal lung specimens were included for this analysis. Our results showed that the expression level of SFN in NSCLC tumor samples was significantly higher than those in adjacent non-neoplastic tissues (p < 0.001; Figure 4B). Furthermore, we analyzed the expression level of SFN from The Cancer Genome Atlas (TCGA) dataset and found that the expression of SFN in NSCLC tumor samples was also significantly higher than that in normal lung tissues (p < 0.001; Figure 4C).

We analyzed the microarray data from 71 NSCLC tumor specimens after ATC, which included 29 NSCLC patients with a poor prognosis (OS status: dead) and 42 NSCLC patients with a good prognosis (OS status: alive) from the GSE14814 dataset, and 49 NSCLC tumor specimens after ATC, which included 24 NSCLC patients with a poor prognosis and 25 NSCLC patients with a good prognosis from the GSE42127 dataset. The results showed that the expression of SFN in NSCLC patients that had a good prognosis was significantly lower than the expression of SFN in NSCLC patients that had a poor prognosis from the GSE14814 dataset (p = 0.017; Figure 4D). Similar results were seen in the expression of SFN in NSCLC patients from the GSE42127 dataset (p = 0.041; Figure 4E). Moreover, the high expression of SFN was significantly associated with decreased OS in NSCLC patients after ATC from the GSE14814 dataset (p = 0.013; Figure 4F). The high expression of SFN was also positively correlated with poor prognosis in NSCLC patients after ATC from the GSE42127 dataset (p = 0.017; Figure 4G).

**Figure 5:**
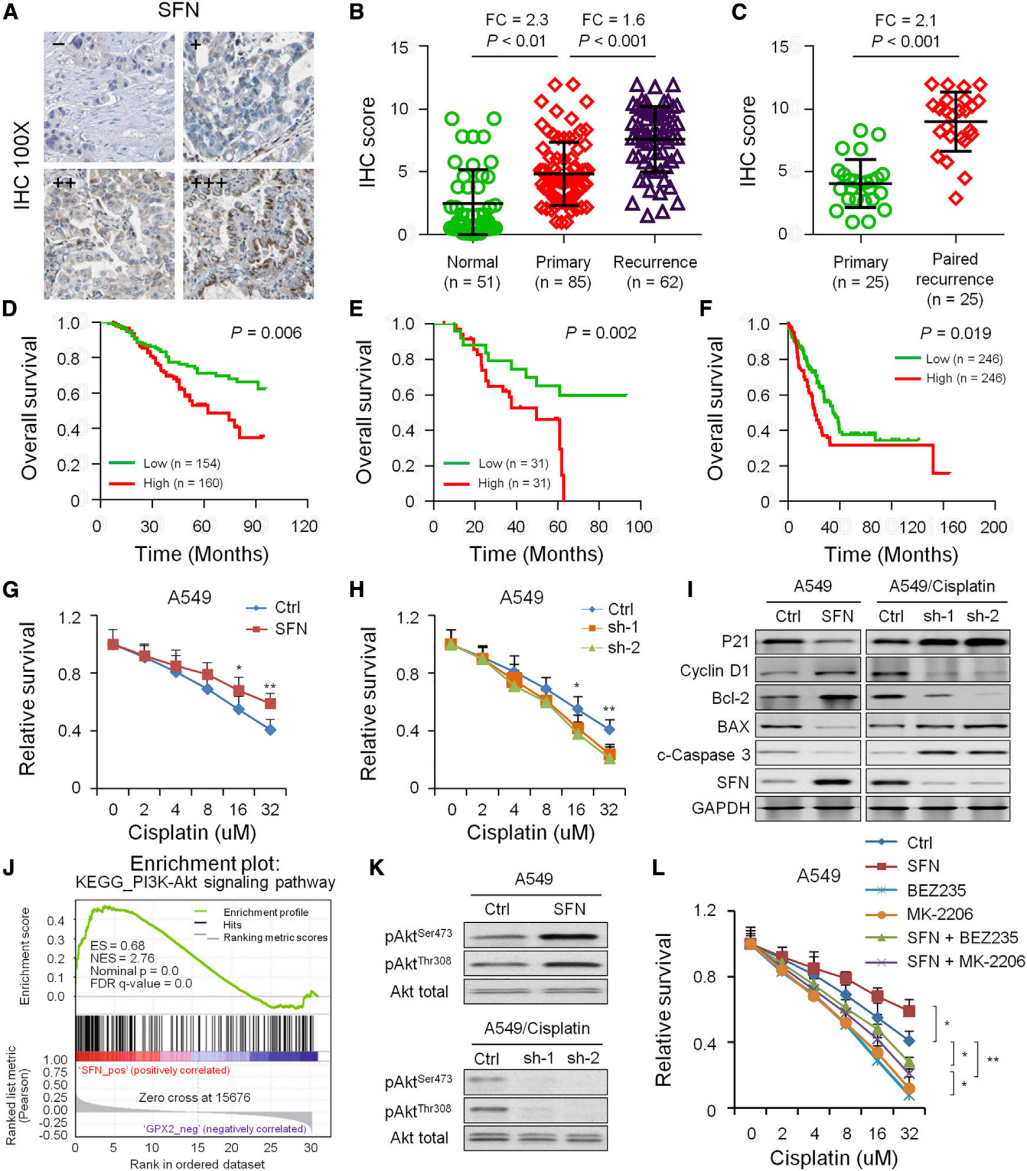
Expression and clinical significance of SFN in normal lung and NSCLC tissues (A) SFN antibody staining of NSCLC tissues. Immunoreactivity scores were defined as the cell staining intensity multiplied by the percentage of stained cells. (B) The expression levels of SFN in adjacent non-cancerous lung tissues (n = 51), primary NSCLC tissue without treatment (n = 85), and recurrent lung cancer tissue after ACT (n = 62). (C) The expression levels of SFN using IHC in 25 paired primary NSCLC tissues and recurrent lung cancer tissues after ACT. (D) Kaplan-Meier survival analysis was used to evaluate the prognostic value of SFN expression for OS in 314 NSCLC patients. (E) Kaplan-Meier survival analysis was used to evaluate the prognostic value of SFN expression for OS in 62 recurrent lung cancer patients after ACT. (F) Kaplan-Meier survival analysis was used to evaluate the prognostic value of SFN expression for OS in 492 LUAD patients from TCGA dataset. (G and H) CCK-8 assays evaluating the drug susceptibility of human lung adenocarcinoma A549 cells to cisplatin after overexpression (G) or knockdown (H) of SFN. (I) Western blotting was performed to detect the associations of SFN expression with proteins of the apoptosis pathway. (J) GSEA analysis was performed to detect the associations of SFN expression with PI3K-Akt pathway activation. (K) Western blotting detected the associations of SFN expression with proteins of the PI3K/Akt pathway. (L) CCK-8 assays evaluating the drug susceptibility of A549 cells after treatment of BEZ235, a dual PI3K/mTOR inhibitor, or MK-2206, an AKT1/2/3 inhibitor.

Results of immunohistochemistry (IHC; Figure 5A) and expression analysis demonstrated that the expression of SFN in primary tumor tissue from NSCLC patients was significantly higher than that in adjacent non-tumor tissues (p < 0.01; Figure 5B). The expression of SFN in recurrent NSCLC patients after ATC was significantly higher than in primary tumor tissues (p < 0.001; Figures 5B and 5C). Moreover, NSCLC patients with high SFN expression levels had poor OS rates as compared to NSCLC patients with low SFN expression (p = 0.006; Figure 5D) (Table 1). High expression of SFN was also positively correlated with poor prognosis in 62 NSCLC patients after ATC (p = 0.002; Figure 5E) and in 492 LUAD patients (p = 0.019; Figure 5F) from TCGA dataset.

**Table 1:** Cox regression model analysis for prognosis based on NSCLC patient clinicopathological characteristics

Factor	HR	95% confidence interval [CI] (univariate)	p value	miR-32 multivariate analysis		
				**HR**	**95% CI (multivariate)**	**p value**

Gender	0.86	0.81–1.12	0.34			
Age	1.08	0.72–1.16	0.46			
Tumor differentiation	1.19	0.94–1.38	0.23			
Diameter	1.98	1.19–3.05	0.012	3.46	2.17–5.32	<0.001
Number of foci	4.72	2.91–5.14	<0.001	4.63	2.20–9.76	<0.001
SFN expression	2.43	1.68–4.33	0.001			

We next attempted to elucidate whether SFN confers cisplatin resistance to NSCLC cells. The results showed that overexpression of SFN attenuated the drug susceptibility of human lung adenocarcinoma A549 cells to cisplatin in cell counting kit-8 (CCK-8) assays (Figure 5G), whereas knockdown of SFN rescued the resistance of A549 cells to cisplatin (Figure 5H). This suggested that SFN may confer chemoresistance in NSCLC. SFN overexpression in A549 cells induced an increase in Cyclin D1 and Bcl-2 expression, and it inhibited p21, Bax, and cleaved caspase-3 expression. However, SFN knockdown in A549/DDP cells caused the opposite effect (Figure 5I).

Gene-set enrichment analysis (GSEA) was performed to explore the potential mechanisms of SFN that promote cisplatin resistance. We found that high expression of SFN was positively correlated with the phosphatidylinositol 3 kinase/v-akt murine thymoma viral oncogene (PI3K/Akt) signaling pathway gene set (p = 0, FDR = 0; Figure 5J). The activation of PI3K/Akt signaling was reported to confer resistance to cisplatin therapy in NSCLC.

To analyze whether the PI3K/Akt pathway was involved in the SFN-induced cisplatin resistance of A549/DDP cells, we performed western blotting. As displayed in Figure 2K, the activation of Akt in SFN overexpressing cells was higher than that in A549 cells, and the level of phosphorylated Akt was significantly decreased after knockdown of SFN. Moreover, treatment with BEZ235, a dual PI3K/mTOR inhibitor, or MK-2206, an AKT1/2/3 inhibitor, rescued the resistance of A549 cells to cisplatin (Figure 5L), suggesting that the activation of the PI3K/Akt pathway and SFN upregulation were associated with cisplatin resistance in A549 cells.

## Discussion

The *SFN* gene encodes a cell cycle checkpoint protein that binds to translation and initiation factors and functions as a regulator of mitotic translation.^5^, ^6^, ^7^, ^8^ SFN has been reported as an oncogene in several cancers, including ovarian cancer and NSCLC.^9^, ^10^, ^11^ However, it is not clear how it influences drug sensitivity in NSCLC patients.

Immunotherapy has revolutionized lung cancer treatments within the past decade, and resistance to immunotherapy, which manifests as a lack of initial response or clinical benefit to therapy or tumor progression after the initial period of response, is frequent.^12^, ^13^, ^14^ Overcoming immunotherapy resistance is challenging because of the complex and dynamic interplay among malignant cells and the immune defense system.^15^, ^16^, ^17^, ^18^

In this study, our results provide the first evidence that the EIF2B4-SFN fusion and increased SFN expression, which activate the PI3K/Akt pathway, are associated with chemotherapy tolerance and poor prognosis in NSCLC patients, suggesting that SFN has potential prognostic value as a molecular marker to identify patients who can possibility benefit from combined treatment. Furthermore, our results suggested that SFN was a potential prognostic marker in lung adenocarcinoma patients after ACT.

## Materials and methods

### Ethics statement

This study was approved by Ethics Committee of Shanghai Tenth People’s Hospital, Tongji University School of Medicine (SHSY-IEC-PAP-15-18). All patients provided their written informed consent.

### Clinical tissue samples

NSCLC and matched non-carcinoma tissue specimens from patients who underwent surgical treatment between 2010 and 2016 were obtained from the tissue bank, Shanghai Tenth People’s Hospital, Tongji University School of Medicine. Two independent, experienced pathologists confirmed diagnosis of NSCLC pathologically. Specimens included paired frozen tumor and adjacent non-cancerous tissues (n = 76) together with a great large number of individual NSCLC frozen biopsies (n = 238). In this cohort, 161 patients are LUADs and 153 patients are LUSCs. Additional 62 paraffin samples of LUAD tissues relapsed after cisplatin-based ACT was collected. Histological typing of the cancers was completed according to the World Health Organization criteria.^19^ Staging was conducted according to the Seventh Edition of the American Joint Commission on Cancer (AJCC) tumor-node-metastasis (TNM) staging system for NSCLC.^20^

### Patients’ clinical information and follow up

The demographic and clinicopathological characteristics were documented including the patient’s clinical information, tumor characteristics, OS, and DFS; patients data were collected up to December 31, 2018 included for all patients. OS was computed in months from the date of diagnosis to the time of death, regardless of cause, and DFS was defined as the time from surgery to the first event of either disease recurrence or death due to any cause.^21^

### Set-up of server for online survival calculation

Two datasets were used for gene expression analysis between normal tissues and cancer tissues: the gene expression profiling from 133 frozen JBR.10 tumor samples were analyzed using GEO datasets, GEO series GSE14814, in which 62 lung cancer samples without chemotherapy treatment (observation, OBS) and 71 lung samples under adjuvant cisplatin/vinorelbine were compared; the primary tumor tissues from 176 patients were selected for GEO datasets, GEO series GSE42127, in which 133 patients are adenocarcinomas (ADCs) and 43 patients are squamous cell carcinomas (SCCs). 49 patients received ACT (mainly Carboplatin plus Taxanes) and 127 patients did not receive ACT. Clinical data are from two GEO datasets including patients’ age, gender, TNM stage, pathologic type, chemotherapy, OS, and DFS.

We downloaded RNA-sequencing data from 515 LUAD and 503 LUSC patients from TCGA portal (https://cancergenome.nih.gov/). The mRNA expression levels were investigated in NSCLC tissues and normal lung tissue tissues in TCGA datasets by Illumina HiSeq 2000 RNA Sequencing Version 2 analysis and normalized by the RSEM algorithm. The clinical information recorded, including the patient’s characteristics, tumor characteristics, and OS and progression-free survival, was assessed.

### RNA isolation and sequencing assay

Three specimens from a NSCLC patient with recurrence (comprising a triplet set of adjacent non-cancerous lung tissue, primary NSCLC, and recurrent lung cancer tissue after ACT) were obtained for RNA sequencing analysis. Total RNA was extracted using TRIzol reagent according to the Kit’s instructions.^22^ RNA concentration was measured in a spectrophotometer, and the quality of all RNA samples was assessed by electrophoresis on 1.5% denaturing Agarose gels.^23^ The mRNA libraries were separately generated from total RNA and constructed according to the standard Illumina RNA library preparation protocol (Illumina, USA).^24^ Sequencing was performed on the Illumina Nextseq 500 platform according to the manufacturer’s instructions. Images generated by Nextseq 500 were converted into nucleotide sequences using a base call pipeline and stored in FASTQ format, and the raw reads were filtered prior to analyzing the data. Clean reads were mapped to reference *Homo sapiens* transcriptome sequences from the UCSC website (hg19) using Bowtie2 and Tophat 2.0.1 software.^25^

### Validation of gene fusion by PCR and Sanger sequencing

The reliability of the gene fusion analysis was assessed using PCR and Sanger sequencing. PCR was performed using the GeneAmp PCR System 9700 (Applied Biosystems, Foster City, CA, USA). About 20 ng template cDNA from each sample was used per reaction. Sequences of PCR primers for EIF2B4-SFN fusion gene are as follows: Forward, 5′-GGCAGATGGTGTGGTATAAC-3; Reverse 1, 5′-CAGAGAACTGCCAGAATCGGG-3′; Reverse 2, 5′-GTTCGGCCTTACTCCGACC-3′. The products were sequenced, and all sequences were analyzed with the Sequencing Analysis Software Version 5.2 (Applied Biosystems).

### qPCR

Total RNA from 76 normal lung tissue and 314 NSCLC samples was isolated with Trizol reagent (Life Technologies, Grand Island, NY, USA). RNA concentration and purity were measured similar to above. qPCR was performed using the TaqMan universal PCR Kit (Life Technologies).^26^ Sequences of PCR primers for SFN gene expression are as follows: Forward, 5′-CACAACACCGCCTAATGAAGA-3; Reverse, 5′-TGCTCGAAGTTCTGACTTGGC-3′; GAPDH were used as the endogenous controls, and the 2^-ΔΔCT^ method was used to analyze expression levels.^27^

### H&E and IHC

Standard IHC and H&E staining were used to evaluate SFN expression levels in 51 normal lung tissues, 85 NSCLC samples without treatment, and 62 NSCLC recurrent samples after ATC.^28^ Primary antibodies against SFN (clone ab14123, dilution 1:500, Cambridge, MA, USA) were included. Immunoreactivity scores were defined as the cell staining intensity (0, nil; 1, weak; 2, moderate; and 3, strong) multiplied by the percentage of stained cells (0, < 5%; 1, 5%–25%; 2, 26%–50%; 3, 51%–75%; 4, 76%–100%), leading to scores from 0 to 12. IHC-positive controls included gene rearranged lung tumor confirmed by PCR and Sanger sequencing. Negative controls included normal liver tissue. Serial sections were stained in parallel with the primary antibody replaced by PBS as mocks.

### Cell culture and stable transfectants

The human LUAD cell line A549 was purchased from Shanghai Institutes for Biological Sciences, Chinese Academy of Cell Resource Center. Cells were cultured at 37°C with 5% CO_2_ in RPMI 1640 medium (HyClone, Logan, UT, USA) with 10% fetal bovine serum (FBS) and 1% penicillin/streptomycin.

For the cell viability assays, A549 cells (3,000 cells per well) were seeded into 96-well culture plates in triplicate and incubated for 5 days at 37°C in a humidified incubator with 5% CO_2_. Every 24 h interval, 10 μL of CCK-8 (Dojindo Laboratories, Japan) was added to each well and incubated at 37°C for 1 h. Then the plates were read at 450 nm (SpectraMax M5, Molecular Devices, USA). All these experiments were repeated at least three times.

The cell line stably expressing SFN was generated by the retroviral infection of A549 cells. Briefly, a retroviral vector containing human SFN cDNA with an N-terminal FLAG-tag, vector for constitutive expression of VSV-G glycoprotein (pHCMV-G), and pCMV-dR8.9 were co-transfected into 293T cells, and the viral supernatants were collected to infect A549 cells.^29^ Monoclonal cells were then selected, cloned, and screened for SFN expression.

SFN-knockdown cell lines were generated using short hairpin RNAs (shRNAs) and retroviral transduction. The lentivirus-mediated SFN shRNA were co-transfected with packaging system using Lipofectamine 3000 (Thermo Fisher Scientific) in HEK293T cells according to the manufacturer’s protocol.^30^ A549 cells were infected with lentiviral particles in the presence of 8 μg/mL polybrene (Sigma-Aldrich, St. Louis, MO, USA) and selected with 1.5 μg/mL puromycin (Sigma-Aldrich). shRNA a random 122 sequence was set up as a control.

### RNA sequencing

RNA isolation, cDNA library construction, and RNA sequencing were performed by Genergy Bio Company (Shanghai, China).^31^ Briefly, total RNA was extracted from A549 and A549/DDP cells using TRIzol reagent (Thermo Fisher Scientific, Waltham, MA, USA), and the quality of extracted RNA was assessed by a Bioanalyzer (Agilent, Waldbronn, Germany). Total RNA samples were treated with DNase I to remove potential genomic DNA, and the polyadenylated fraction of RNA was isolated for RNA-seq library preparation. A TruSeq Stranded mRNA sample prep kit (Illumina, San Diego, CA, USA) was used to construct the stranded libraries by following the manufacturer’s instructions. All libraries were sequenced on an Illumina HiSeq 2500 sequencer (Novogene Bioinformatics Technology, Beijing, China). Genes that were differentially expressed between A549 and A549/DDP cells were identified by DESeq2 R package.^31^ According to the results of DESeq2 analysis, genes were classified as differentially expressed when fold changes were more than two and the statistically calculated p value was less than 0.05. Three replicates were tested for each cell line.

### GSEA

GSEA was performed by using a JAVA program with MSigDB C2 CP: canonical pathways gene set collection.^32^ First, based on their correlation with SFN expression, GSEA generated an ordered list of all genes and then a predefined gene set (signature of gene expression upon perturbation of certain cancer-related gene) received an enrichment score (ES), which is a measure of statistical evidence rejecting the null hypothesis that the members of the list are randomly distributed in the ordered list. Parameters used for the analysis were as follows: “c2.all.v5.0.symbols. gmt” gene sets to run GSEA, 1,000 permutations to calculate p value, and the permutation type was set to gene set. The maximum gene set size was fixed at 1,500 genes, while the minimum size fixed at 15 genes. SFN expression level was used as phenotype label, and “Metric for ranking genes” was set to Pearson correlation. All other basic and advanced fields were set to default.

### Western blot analysis

Following treatment with the selected compounds, the cells were collected and lysed in radioimmunoprecipitation assay (RIPA) buffer containing protease and phosphatase inhibitors. Protein concentrations were quantified using the BCA kit. Standard western blot was used to evaluate total and phosphorylated protein expression levels. The antibodies used in this study were as follows: anti-Akt (1:1,000; AbSci, cat. no. AB 4685), anti-phospho-Akt (1:1,000; Ser473, AbSci, cat. no. AB 4060; Thr308, AbSci, cat. no. AB11055), anti-pro caspase-3 (1:1,000; AbSci, cat. no. AB9665), and p21 (1:1,000; Abcam, ab218311), Cyclin D1 (1:1,000; Abcam, ab134175), Bcl-2 (1:1,000; Abcam, ab185002), Bax (1:1,000; Abcam, ab32503), cleaved caspase 3 (1:1,000; Abcam, ab32042), and GAPDH (1:1,000; Abcam, ab181602).

### Bioinformatic analysis

Microarray hierarchical clustering for transcriptome analysis regroups the patients according to the similarities of their biological profiles and identifies the combinations of variables classifying the patients. For each patient and each biological variable, the value determined for one patient is normalized as a function of the median of all the values obtained from this population. Hierarchical clustering was performed using the multiple experiment viewer (MeV) 4.7.1 software (https://mev.tm4.org/).^33^ Gene ontology (GO) classifications was used (http://www.geneontology.org/) to evaluate the biological function of the changed genes in NSCLC through three aspects including biological process, molecular function, and cellular components.^34^ Subsequently, dysregulated genes were subjected to KEGG pathway analysis.^35^

### Statistical analyses

The statistical software SPSS 23.0 (SPSS, Chicago, IL, USA), and GraphPad Prism 7 (San Diego, CA, USA) were used for the statistical analysis and generation of figures. For all statistical methods, the significance of differences between groups was assessed using Student’s t test or one-way ANOVA. Using the median as cutoff value, patients were divided into those with a high or low score based on the expression levels of SFN. Independent t test was used to evaluate differences between two groups, and chi-square test was used to examine differences in groups. OS and DFS curves were constructed using Kaplan-Meier survival analysis, and results were compared using the log-rank test. In order to estimate independent prognostic factors associated with survival, univariate and multivariate survival analyses were performed using the Cox regression model. All statistical results were presented as mean ± standard deviation (SD); each experiment has three biological repetitions; p < 0.05 was considered as the significant difference.

## References

[1] Wu L., Wen Z., Song Y., Wang L. (2021). A novel autophagy-related lncRNA survival model for lung adenocarcinoma. J. Cell. Mol. Med..

[2] Liu X., Xiang D., Xu C., Chai R. (2021). EIF3m promotes the malignant phenotype of lung adenocarcinoma by the up-regulation of oncogene CAPRIN1. Am. J. Cancer Res..

[3] Chen J., Liu A., Lin Z., Wang B., Chai X., Chen S., Lu W., Zheng M., Cao T., Zhong M. (2020). Downregulation of the circadian rhythm regulator HLF promotes multiple-organ distant metastases in non-small cell lung cancer through PPAR/NF-κb signaling. Cancer Lett..

[4] Pepe C., Hasan B., Winton T.L., Seymour L., Graham B., Livingston R.B., Johnson D.H., Rigas J.R., Ding K., Shepherd F.A., National Cancer Institute of Canada and Intergroup Study JBR.10 (2007). Adjuvant vinorelbine and cisplatin in elderly patients: National Cancer Institute of Canada and Intergroup Study JBR.10. J. Clin. Oncol..

[5] Shiba-Ishii A., Kim Y., Shiozawa T., Iyama S., Satomi K., Kano J., Sakashita S., Morishita Y., Noguchi M. (2015). Stratifin accelerates progression of lung adenocarcinoma at an early stage. Mol. Cancer.

[6] Jia G., Wen W., Massion P.P., Shu X.O., Zheng W. (2021). Incorporating both genetic and tobacco smoking data to identify high-risk smokers for lung cancer screening. Carcinogenesis.

[7] Brown C.N., Atwood D.J., Pokhrel D., Ravichandran K., Holditch S.J., Saxena S., Miyazaki M., Nemenoff R., Weiser-Evans M.C.M., Ljubanovic D.G. (2020). The effect of MEK1/2 inhibitors on cisplatin-induced acute kidney injury (AKI) and cancer growth in mice. Cell. Signal..

[8] Ma Y.S., Liu J.B., Yang X.L., Xin R., Shi Y., Zhang D.D., Wang H.M., Wang P.Y., Lin Q.L., Li W., Fu D. (2021). Basic approaches, challenges and opportunities for the discovery of small molecule anti-tumor drugs. Am. J. Cancer Res..

[9] Quartararo A.J., Gates Z.P., Somsen B.A., Hartrampf N., Ye X., Shimada A., Kajihara Y., Ottmann C., Pentelute B.L. (2020). Ultra-large chemical libraries for the discovery of high-affinity peptide binders. Nat. Commun..

[10] Han X., Yang J., Zeng F., Weng J., Zhang Y., Peng Q., Shen L., Ding S., Liu K., Gao Y. (2020). Programmable Synthetic Protein Circuits for the Identification and Suppression of Hepatocellular Carcinoma. Mol. Ther. Oncolytics.

[11] Lv H.W., Xing W.Q., Ba Y.F., Li H.M., Wang H.R., Li Y. (2021). SMYD3 confers cisplatin chemoresistance of NSCLC cells in an ANKHD1-dependent manner. Transl. Oncol..

[12] Wang X., Qiao G., Jiang N., Morse M.A., Zhou X., Wang S., Wu J., Song Y., Zhao Y., Zhou L. (2021). Serial assessment of circulating T lymphocyte phenotype and receptor repertoire during treatment of non-muscle invasive bladder cancer with adoptive T cell immunotherapy. Am. J. Cancer Res..

[13] Yang Y., Yang J., Shen L., Chen J., Xia L., Ni B., Ge L., Wang Y., Lu S. (2021). A multi-omics-based serial deep learning approach to predict clinical outcomes of single-agent anti-PD-1/PD-L1 immunotherapy in advanced stage non-small-cell lung cancer. Am. J. Transl. Res..

[14] Wei G., Zhang H., Zhao H., Wang J., Wu N., Li L., Wu J., Zhang D. (2021). Emerging immune checkpoints in the tumor microenvironment: Implications for cancer immunotherapy. Cancer Lett..

[15] Zimmerman K.A., Hopp K., Mrug M. (2020). Role of chemokines, innate and adaptive immunity. Cell. Signal..

[16] Ni W., Mo H., Liu Y., Xu Y., Qin C., Zhou Y., Li Y., Li Y., Zhou A., Yao S. (2021). Targeting Cholesterol Biosynthesis Promotes Anti-tumor Immunity by Inhibiting Long Noncoding RNA SNHG29 Mediated YAP Activation. Mol. Ther..

[17] Chong W., Wang Z., Shang L., Jia S., Liu J., Fang Z., Du F., Wu H., Liu Y., Chen Y., Chen H. (2020). Association of clock-like mutational signature with immune checkpoint inhibitor outcome in patients with melanoma and NSCLC. Mol. Ther. Nucleic Acids.

[18] Labani-Motlagh A., Naseri S., Wenthe J., Eriksson E., Loskog A. (2021). Systemic immunity upon local oncolytic virotherapy armed with immunostimulatory genes may be supported by tumor-derived exosomes. Mol. Ther. Oncolytics.

[19] Deng F., Shen L., Wang H., Zhang L. (2020). Classify multicategory outcome in patients with lung adenocarcinoma using clinical, transcriptomic and clinico-transcriptomic data: machine learning versus multinomial models. Am. J. Cancer Res..

[20] Lantuejoul S., Rouquette I., Brambilla E., Travis W.D. (2016). [New WHO classification of lung adenocarcinoma and preneoplasia]. Ann. Pathol..

[21] Ma Y.S., Liu J.B., Lin L., Zhang H., Wu J.J., Shi Y. (2021). Exosomal microRNA-15a from mesenchymal stem cells impedes hepatocellular carcinoma progression via downregulation of SALL4. Cell Death Discov..

[22] Han F., Yang S., Wang W., Huang X., Huang D., Chen S. (2020). Silencing of lncRNA LINC00857 Enhances BIRC5-Dependent Radio-Sensitivity of Lung Adenocarcinoma Cells by Recruiting NF-κB1. Mol. Ther. Nucleic Acids.

[23] Wang F., Quan Q. (2020). The long non-coding RNA SNHG4/microRNA-let-7e/KDM3A/p21 pathway is involved in the development of non-small cell lung cancer. Mol. Ther. Oncolytics.

[24] Cefalo C.M.A., Mezza T., Giaccari A., Kulkarni R.N. (2021). A Systematic Comparison of Protocols for Recovery of High-Quality RNA from Human Islets Extracted by Laser Capture Microdissection. Biomolecules.

[25] Ghosh S., Chan C.K. (2016). Analysis of RNA-Seq Data Using TopHat and Cufflinks. Methods Mol. Biol..

[26] Ma Y.S., Cao Y.F., Liu J.B., Li W., Deng J., Yang X.L. (2021). The power and the promise of circRNAs for cancer precision medicine with functional diagnostics and prognostic prediction. Carcinogenesis.

[27] Livak K.J., Schmittgen T.D. (2001). Analysis of relative gene expression data using real-time quantitative PCR and the 2^(-Δ^^Δ C(T^)) Method. Methods.

[28] Narayanapillai S.C., Han Y.H., Song J.M., Kebede M.E., Upadhyaya P., Kassie F. (2020). Modulation of the PD-1/PD-L1 immune checkpoint axis during inflammation-associated lung tumorigenesis. Carcinogenesis.

[29] Yang B., Zhao F., Yao L., Zong Z., Xiao L. (2021). CircRNA circ_0006677 Inhibits the Progression and Glycolysis in Non-Small-Cell Lung Cancer by Sponging miR-578 and Regulating SOCS2 Expression. Front. Pharmacol..

[30] Kim T., Viard M., Afonin K.A., Gupta K., Popov M., Salotti J., Johnson P.F., Linder C., Heldman E., Shapiro B.A. (2020). Characterization of Cationic Bolaamphiphile Vesicles for siRNA Delivery into Tumors and Brain. Mol. Ther. Nucleic Acids.

[31] Varet H., Brillet-Guéguen L., Coppée J.Y., Dillies M.A. (2016). SARTools: A DESeq2- and EdgeR-Based R Pipeline for Comprehensive Differential Analysis of RNA-Seq Data. PLoS ONE.

[32] Subramanian A., Kuehn H., Gould J., Tamayo P., Mesirov J.P. (2007). GSEA-P: a desktop application for Gene Set Enrichment Analysis. Bioinformatics.

[33] Traver G., Sekhar K.R., Crooks P.A., Keeney D.S., Freeman M.L. (2021). Targeting NPM1 in irradiated cells inhibits NPM1 binding to RAD51, RAD51 foci formation and radiosensitizes NSCLC. Cancer Lett..

[34] Wang H.L., Liu P.F., Yue J., Jiang W.H., Cui Y.L., Ren H., Wang H., Zhuang Y., Liu Y., Jiang D. (2020). Somatic gene mutation signatures predict cancer type and prognosis in multiple cancers with pan-cancer 1000 gene panel. Cancer Lett..

[35] Chen Y., Zhao H., Li H., Feng X., Tang H., Qiu C., Zhang J., Fu B. (2020). LINC01234/MicroRNA-31-5p/MAGEA3 Axis Mediates the Proliferation and Chemoresistance of Hepatocellular Carcinoma Cells. Mol. Ther. Nucleic Acids.

